# Physiological and metabolic responses of *Anabaena variabilis* and *Chlorella pyrenoidosa* under lead stress: implications for microalgal bioremediation

**DOI:** 10.3389/fbioe.2026.1842216

**Published:** 2026-05-07

**Authors:** Kholoud K. Alzahrani

**Affiliations:** Department of Biology, University College of Umluj, University of Tabuk, Tabuk, Saudi Arabia

**Keywords:** *Anabaena variabilis*, antioxidant enzymes, bioremediation, *Chlorella pyrenoidosa*, lead contamination, oxidative stress

## Abstract

Lead (Pb) contamination represents a substantial threat to aquatic ecosystems, and phototrophic microorganisms are increasingly regarded as promising agents for sustainable bioremediation. While previous studies have established algal biosorption capabilities, the unique contribution of this work lies in providing a comprehensive comparative framework between taxonomically distinct species to elucidate diverging, species-specific Pb detoxification strategies. In this study, the physiological, biochemical, and metabolic responses of the cyanobacterium *Anabaena variabilis* and the green microalga *Chlorella pyrenoidosa* were evaluated following 4 days of exposure to 0, 300, and 500 μg L^-1^ Pb using inductively coupled plasma mass spectrometry and comprehensive biochemical profiling. In *A. variabilis*, Pb accumulation was highly efficient at the moderate 300 μg L^-1^ exposure (sequestering 135.57 μg g^-1^) and 237.70 μg g^-1^ at 500 μg L^-1^. This metal burden sequentially compromised its photosynthetic capacity, driving a progressive decline in chlorophyll *a* content, Rubisco activity, and CO_2_ fixation. Concurrently, Pb induced moderate oxidative stress, evidenced by elevated H_2_O_2_ levels, lipid peroxidation, and protein oxidation. To cope, *A. variabilis* upregulated antioxidant enzymes, particularly superoxide dismutase, and initiated metabolic adjustments such as the accumulation of soluble sugars. However, these defenses were insufficient to fully mitigate oxidative damage at the highest concentration. Conversely, *C. pyrenoidosa* initially sequestered substantially less Pb at 300 μg L^-1^ (52.89 μg g^-1^) but demonstrated a massive surge in intracellular accumulation at 500 μg L^-1^ (205.09 μg g^-1^). At this elevated concentration, *C. pyrenoidosa* exhibited significantly greater photosynthetic susceptibility and severe oxidative stress, marked by a sharp increase in NADPH oxidase activity. To survive this severe toxicity, the microalga deployed extreme metabolic plasticity. This response was characterized by the robust activation of catalase, ascorbate peroxidase, glutathione, and ascorbate, alongside profound metabolic reprogramming involving the accumulation of glycine, specific organic acids, and extensive remodeling of its fatty acid composition. In a nutshell, *A. variabilis* demonstrated a higher Pb accumulation capacity under moderate exposure, whereas *C. pyrenoidosa* displayed marked metabolic plasticity under severe Pb stress. These distinct differences underscore complementary traits that are potentially highly advantageous for designing integrated phycoremediation and biomonitoring applications.

## Graphical Abstract

**Figure:**
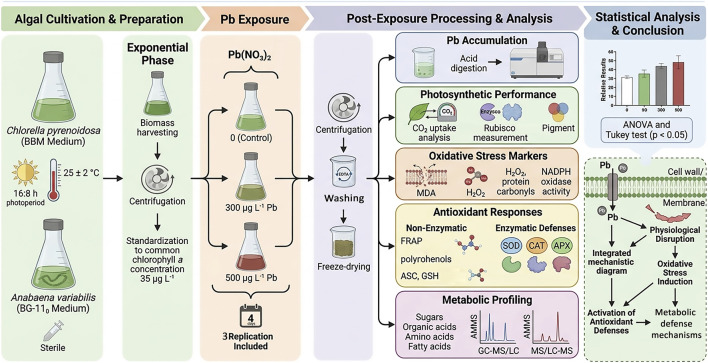
Schematic overview of the experimental design investigating lead (Pb) toxicity in the microalgae *Chlorella pyrenoidosa* and cyanobacterium *Anabaena variabilis*.

## 1 Introduction

The accelerating pace of industrialization and urbanization over the past several decades has resulted in the unprecedented release of heavy metals into aquatic and terrestrial ecosystems, posing severe threats to environmental integrity and public health (Mishra et al., 2025; Tarfeen et al., 2022). Among the most hazardous of these contaminants is lead (Pb), a non-essential, non-biodegradable heavy metal that originates from a wide array of anthropogenic sources including mining, smelting, lead-acid battery manufacturing, electronic waste processing, ceramics production, bangle industries, ship building, road runoff, and the paints industry (Saraswat et al., 2020; Yadav et al., 2025). Once released into the environment, lead persists indefinitely, accumulates in food chains, and exerts toxic effects on virtually all forms of life (Saraswat et al., 2020). In humans, lead exposure has been associated with developmental delays, kidney damage, neurological impairment, and immunosuppression (Ankit and Korstad, 2022). In aquatic ecosystems, elevated lead concentrations elevate the production of reactive oxygen species (ROS), which can cause oxidative damage to fish and other aquatic organisms (Ankit and Korstad, 2022). In plants and algae, lead toxicity manifests as inhibition of photosynthesis, disruption of enzymatic activities, induction of oxidative stress, and impairment of growth and metabolic processes (Huang et al., 2022).

The persistence and bioaccumulative nature of lead, combined with its carcinogenic, teratogenic, and mutagenic properties, have made its remediation a matter of urgent global concern (Seeta and Raghavendra, 2025). Conventional physicochemical approaches for heavy metal removal, including chemical precipitation, reverse osmosis, ion exchange, membrane filtration, and thermal treatment, are often prohibitively expensive, energy-intensive, and generate secondary pollutants that themselves require further management (Murugavelh and Vinothkumar, 2010; Zeraatkar et al., 2016). These limitations have driven the search for more sustainable, cost-effective, and ecologically compatible remediation strategies.

Bioremediation, broadly defined as the use of living organisms or their products to detoxify, remove, or immobilize environmental contaminants, has emerged as a compelling alternative to conventional physicochemical methods (Alharbi et al., 2025; Tarfeen et al., 2022). Among the diverse array of biological agents investigated for heavy metal remediation, algae encompassing both eukaryotic microalgae and prokaryotic cyanobacteria have attracted particular attention due to their unique combination of biochemical, physiological, and ecological properties (Ankit and Korstad, 2022). The use of algae for environmental remediation, termed phycoremediation, exploits the capacity of algal biomass to sequester, accumulate, and detoxify heavy metals, including lead from contaminated water and effluents (Fathy et al., 2026; Igiri et al., 2018). Algae are considered superior biosorbents compared to many other microbial agents for several compelling reasons. They are autotrophic organisms requiring minimal nutrient inputs, capable of producing large quantities of biomass while simultaneously fixing atmospheric CO_2_ (Igiri et al., 2018; Zeraatkar et al., 2016). Their cell walls are rich in polysaccharides, proteins, and lipids bearing functional groups including hydroxyl, carboxyl, phosphate, amide, sulfate, and amino groups that serve as active metal-binding sites (Igiri et al., 2018; Kharel et al., 2023). Algal biosorption effectiveness has been reported to be approximately 15.3%–84.6% higher than that of other fungi and bacteria (Ankit and Korstad, 2022). For example, exposure of *S. obliquus* to chromium contamination has been reported to result in metal removal efficiencies exceeding 90%. Similarly, *Chlorella* sp. Has demonstrated the capacity to precipitate more than 80% of cadmium ions from aqueous solution (Parmar and Patel, 2025). Therefore, the integration of algal bioremediation with wastewater treatment and bioenergy production offers additional economic and environmental benefits, creating a circular bioeconomy model (Raikova et al., 2019; Zeraatkar et al., 2016). Despite growing interest in algal phycoremediation, species differ markedly in their tolerance, uptake behavior, and biochemical defense capacity under metal stress. A comparative assessment using taxonomically distinct organisms, such as a green microalga and a cyanobacterium, can therefore improve understanding of Pb detoxification strategies and help identify strains with better remediation potential. Accordingly, the present study examined Pb accumulation and its concomitant impacts using a multi-tiered suite of biomarkers. This particular assemblage of indicators encompassing photosynthesis-related traits, oxidative stress markers, antioxidant defense components, and central metabolic regulators (including sugars, amino and organic acids, and fatty acids) was selected to enable a comprehensive, systems-level evaluation of both primary metal-induced toxicity and secondary systemic acclimation responses in *A*. *variabilis* and *C*. *pyrenoidosa* subjected to Pb exposure under controlled laboratory conditions.

## 2 Materials and methods

### 2.1 Strains and culture conditions

The algal strains *C*. *pyrenoidosa* and *A*. *variabilis* were procured from Algae Research and Supply Co. (San Diego, CA, USA). *Chlorella pyrenoidosa* was maintained in Bold’s Basal Medium (BBM) (Bischoff, 1963), whereas *A. variabilis* was cultured in BG-11_0_ medium Stanier et al. (1971). All cultures were grown photo-autotrophically in an orbital shaker incubator at 120 rpm and 25 °C ± 2 °C. Illumination was supplied at an intensity of 75 µmol photons m^-2^ s^-1^ under a 16 h light/8 h dark photoperiod. These experimental parameters were selected to approximate the natural diurnal irradiance regime and light environment characteristic of shallow aquatic ecosystems during periods of active primary production. Moreover, this moderate photon flux density supplies adequate energy to sustain autotrophic growth and detoxification-related metabolic processes, while minimizing confounding photo-oxidative stress. This design thus enables the specific attribution of observed physiological and biochemical responses to Pb exposure. To minimize trace-metal contamination, all glassware and plasticware were soaked in 10% (v/v) nitric acid for 24 h, thoroughly rinsed with Milli-Q water, and air-dried before use.

### 2.2 Experimental design and Pb exposure

Both strains were initially cultivated in metal-free medium until they reached the exponential growth phase. For each strain, 200 mL of culture was harvested by centrifugation at 5,000 rpm for 15 min under aseptic conditions, and the resulting cell pellets were resuspended in fresh medium. The resuspended biomass was subsequently transferred into sterile 500 mL Erlenmeyer flasks containing 300 mL of fresh culture medium, and the pH was adjusted to 6.8. Lead exposure experiments were initiated by adding Pb, supplied as Pb(NO_3_)_2_, to obtain final concentrations of 0 (control), 300, and 500 μg L^-1^. Whereas these concentrations were deliberately selected to represent environmentally relevant levels of Pb contamination commonly reported in polluted aquatic ecosystems and in water bodies impacted by industrial or agricultural effluents. This concentration range is intended to emulate realistic ecological stress conditions rather than severe acute toxicity, thereby enabling the evaluation of natural metabolic acclimation processes. The initial biomass concentration in each flask was standardized to a chlorophyll *a* concentration of 35 μg L^-1^. Each treatment comprised three independent replicate cultures. Cultures were incubated for 4 days under the growth conditions described above. This exposure period was selected because it is consistent with the standardized ecotoxicological test protocol 201 guidelines of OECD (2011) for the evaluation of acute physiological, biochemical, and growth responses in microalgae during their exponential growth phase, thereby minimizing confounding effects associated with culture senescence or extensive cell mortality. At the end of the exposure period, cultures were harvested by centrifugation at 5,000 rpm for 15 min. The resulting biomass pellets were washed twice with Pb-free medium to remove loosely surface-associated metal ions, and then the pellets were subsequently rinsed with Milli-Q water. The washed biomass was frozen at −80 ∘C and lyophilized for 24 h. The resulting dried samples were stored in acid-washed polypropylene tubes until further analysis.

### 2.3 Determination of Pb uptake

Lead accumulation in the strains biomass was quantified following microwave-assisted acid digestion and subsequent determination by inductively coupled plasma mass spectrometry (ICP-MS), conducted in accordance with the guidelines of EPA Method 3,052 to ensure complete dissolution of biological and organic matrices (US-EPA, 1996). For each determination, 30 mg of freeze-dried biomass were transferred to a microwave digestion vessel and digested with 10 mL of concentrated HNO_3_ and 2 mL of HCl. After digestion, the samples were allowed to cool, filtered, transferred to 25 mL volumetric flasks, and made up to volume with Milli-Q water. Before ICP-MS measurement, digests were diluted 1:50 with Milli-Q water. Calibration standards were prepared from a 1 mg L^-1^ Pb(NO_3_)_2_ stock solution. To ensure analytical accuracy and precision, a NIST-traceable certified reference material was analyzed in parallel with the algal samples. In addition, matrix spike recovery experiments were conducted by fortifying blank biomass with known concentrations of Pb before digestion. The resulting recoveries ranged from 94% to 102%, with a relative standard deviation of less than 5% across analytical triplicates. Procedural blanks and quality-control standards were included in each analytical batch to assess and ensure accuracy and precision. The limits of detection and quantification for Pb were calculated from the standard deviation of the blanks and were typically in the low μg L^-1^ range.

### 2.4 Physiological responses and photosynthetic performance

#### 2.4.1 Pigment quantification

Chlorophyll *a* and carotenoids were quantified as proxies for the structural and functional integrity of the photosynthetic apparatus. Briefly, 1 mL aliquots of each culture were centrifuged at 5,000 rpm for 15 min to obtain cell pellets. Pigments were extracted from the pellets with 5 mL of 80% (v/v) acetone. The absorbance of the pigment extracts was measured spectrophotometrically at 452, 650, and 665 nm using 80% (v/v) acetone as the blank, enabling the quantitative determination of both pigment classes.

#### 2.4.2 Carbon assimilation and rubisco activity

Photosynthetic performance was assessed by quantifying net CO_2_ assimilation following the protocol established by Hällbom and Bergman (1983). In parallel, the specific enzymatic activity of ribulose-1,5-bisphosphate carboxylase/oxygenase (Rubisco) was determined. Biomass from each treatment was harvested and pelleted by centrifugation (5,000 rpm, 30 min, 4 °C), immediately snap-frozen in liquid nitrogen, and subsequently homogenized in a standard HEPES/KOH extraction buffer (pH 7.5) supplemented with a protease inhibitor cocktail. Rubisco activity was then quantified using a highly sensitive, non-radioactive, microplate-based enzymatic assay that continuously monitors the formation of 3-phosphoglycerate, strictly adhering to the protocol described by Sulpice et al. (2007).

### 2.5 Oxidative stress and antioxidant defense systems

#### 2.5.1 Oxidative damage biomarkers

Oxidative stress in Pb-exposed cells was evaluated by quantifying cellular damage indicators. Malondialdehyde (MDA), used as an index of lipid peroxidation, was determined via the thiobarbituric acid reaction method described by Hodges et al. (1999). Hydrogen peroxide (H_2_O_2_) content was measured using the FOX1 assay, which relies on the peroxide-dependent oxidation of Fe^2+^, following Jiang et al. (1990). Furthermore, protein oxidation was assessed by quantifying carbonyl groups using a colorimetric assay based on derivatization with 2,4-dinitrophenylhydrazine (DNPH), according to the procedure of Levine (1994).

#### 2.5.2 ROS-generating and antioxidant enzymes

The activities of NADPH oxidase and the antioxidant enzymes ascorbate peroxidase (APX), superoxide dismutase (SOD), and catalase (CAT) were quantified to assess enzymatic defenses. Freeze-dried biomass was homogenized in a standard potassium phosphate extraction buffer (pH 7.0) supplemented with stabilizers and protease inhibitors. Specific enzyme activities were measured spectrophotometrically based on established protocols: NADPH oxidase according to Sarath et al. (2007); APX following the method of Murshed et al. (2008); SOD via the inhibition of nitro-blue tetrazolium (NBT) reduction at 560 nm (Dhindsa et al., 1981); and CAT via the continuous decomposition of H_2_O_2_ at 240 nm (Aebi, 1984).

#### 2.5.3 Non-enzymatic antioxidants

To evaluate non-enzymatic defense mechanisms, the contents of the antioxidant metabolites ascorbate (ASC) and reduced glutathione (GSH) were extracted from the biomass. These metabolites were successfully separated and quantified using high-performance liquid chromatography (HPLC) equipped with UV-Vis detection and a reverse-phase C18 column, strictly following the protocol described by Potters et al. (2004).

### 2.6 Metabolic and biochemical profiling

#### 2.6.1 Soluble carbohydrates

To evaluate the reallocation of energy resources under Pb stress, soluble carbohydrates were extracted from the microalgal cultures using 80% (v/v) ethanol. The extracts were dried, reconstituted in deionized water, and analyzed using an HPLC system equipped with a diode array detector. Specific sugar concentrations were determined by external calibration against authentic glucose, fructose, and sucrose standards.

#### 2.6.2 Organic acids

Organic acids acting as potential metal-chelating agents (e.g., malic, citric, and oxalic acids) were extracted using an acidified solution containing 0.1% phosphoric acid and 0.3% (w/v) butylated hydroxyanisole. Quantification was carried out via HPLC utilizing a SUPELCOGEL C-610H column with ultraviolet detection at 210 nm against corresponding analytical standards.

#### 2.6.3 Amino acids

Specific amino acids implicated in stress acclimation (glycine, glutamic acid, and cystine) were extracted using 80% aqueous ethanol. Following standard purification and filtration steps, the amino acids were separated on a BEH amide column and accurately quantified using a Waters Acquity UPLC-QDa mass spectrometer, strictly following the established method described by Sinha et al. (2013).

#### 2.6.4 Fatty acid composition

To assess lipid remodeling in response to Pb toxicity, fatty acids were extracted from dried microalgal biomass using 50% aqueous methanol. The extracts were subsequently analyzed by gas chromatography-mass spectrometry (GC-MS) equipped with an HP-5 MS column. Individual saturated and unsaturated fatty acids were structurally identified by comparing their mass spectra with the standardized NIST 05 and Golm Metabolome reference databases.

### 2.7 Statistical analysis

All experiments were conducted using three independent biological replicates per treatment in a completely randomized design. Data are presented as mean ± standard deviation (SD). Statistical analyses and graphical visualization were performed using GraphPad Prism (version 10). Differences between Pb treatments for each strain were evaluated using one-way and two-way analysis of variance (ANOVA), followed by Tukey’s multiple comparison *post hoc* test. Statistical significance was defined using a conventional threshold of p < 0.05. To convey the magnitude and gradations of treatment effects with greater resolution, distinct significance levels are indicated in the figures as follows: “ns” denotes non-significant differences (p ≥ 0.05), * denotes p < 0.05, ** denotes p < 0.01, *** denotes p < 0.001, and **** denotes p < 0.0001.

## 3 Results

### 3.1 Lead bioaccumulation dynamics

**FIGURE 1:**
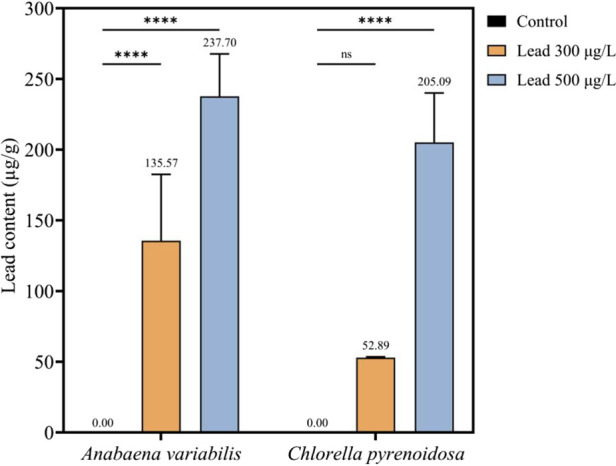
Lead content in the biomass of *Anabaena variabilis* and *Chlorella pyrenoidosa* after 4 days of exposure to 0, 300, and 500 μg L^-1^ Pb (Values represent the mean ± SD of three independent replicates). Different significance levels are indicated in the figure, where “ns” denotes non-significant differences and “****” indicates p < 0.0001.

Lead bioaccumulation in both taxa exhibited a clear dose-dependent response. In *A. variabilis*, Pb content rose from 0 μg g^-1^ at control to 135.57 μg g^-1^ at 300 μg L^-1^, reaching a maximum of 237.70 μg g^-1^ at 500 μg L^-1^. This represents a 75.3% increase in accumulation between the two treatment levels. While *C. pyrenoidosa* followed a similar trend, its response to increased concentration was more pronounced; accumulation surged by 287.8% (from 52.89 to 205.09 μg g^-1^) when the exposure was raised from 300 to 500 μg L^-1^. Notably, the interspecific disparity in Pb uptake narrowed at higher concentrations. Although *A. variabilis* accumulated 2.56-fold more Pb than *C. pyrenoidosa* at 300 μg L^-1^, this margin decreased to 15.9% at 500 μg L^-1^, suggesting a potential saturation of binding sites in *A. variabilis* or a shift in the uptake kinetics of *C. pyrenoidosa* at elevated concentrations (Figure 1).

### 3.2 Photosynthetic performance

#### 3.2.1 Photosynthetic pigments

**FIGURE 2:**
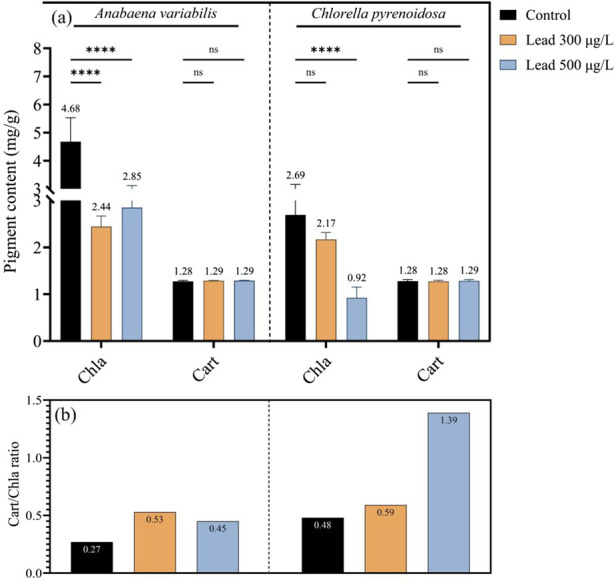
Chlorophyll a (Chla) and carotenoid (Car) contents **(a)** and the carotenoid/chlorophyll a (Car/Chla) ratio **(b)** in *A*. *variabilis* and *C*. *pyrenoidosa* after 4 days of exposure to 0, 300, and 500 μg L^-1^ Pb (Values represent the mean ± SD of three independent replicates). Significance annotations in **(a)** indicate differences among treatments, where “ns” denotes non-significant differences and “****” indicates p < 0.0001.

In *A. variabilis*, chlorophyll *a* concentration declined from 4.68 mg g^-1^ in the control to 2.44 mg g^-1^ at 300 μg L^-1^ Pb and 2.85 mg g^-1^ at 500 μg L^-1^ Pb, corresponding to reductions of 47.9% and 39.1%, respectively, relative to the control. In contrast, carotenoid content showed negligible variation, with values of 1.28, 1.29, and 1.29 mg g^-1^ in the control, 300 μg L^-1^, and 500 μg L^-1^ Pb treatments, respectively. While in *C. pyrenoidosa*, chlorophyll *a* content decreased from 2.69 mg g^-1^ in the control to 2.17 mg g^-1^ at 300 μg L^-1^ Pb and 0.92 mg g^-1^ at 500 μg L^-1^ Pb, representing declines of 19.3% and 65.8%, respectively, compared with the control. Carotenoid content remained essentially constant across all treatments, with measured values of 1.28 mg g^-1^ in the control and 300 μg L^-1^ treatments, and 1.29 mg g^-1^ at 500 μg L^-1^ Pb (Figure 2a). Moreover, the carotenoid-to-chlorophyll *a* ratio in *A. variabilis* increased from 0.27 in the control to 0.53 at 300 μg L^-1^ and 0.45 at 500 μg L^-1^ Pb. In *C. pyrenoidosa*, this ratio increased from 0.48 in the control to 0.59 at 300 μg L^-1^ and further to 1.39 at 500 μg L^-1^ Pb (Figure 2b).

#### 3.2.2 Carbon assimilation (rubisco and CO_2_ fixation)

**FIGURE 3:**
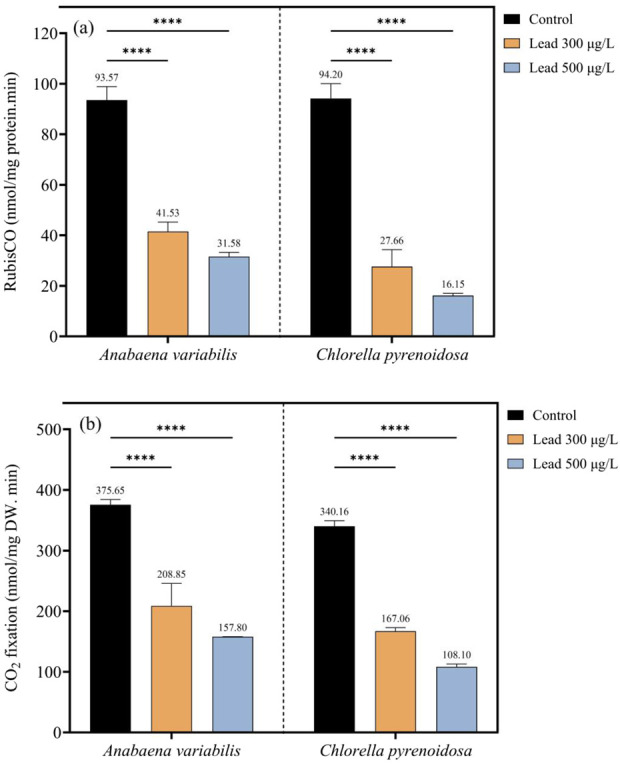
Ribulose-1,5-bisphosphate carboxylase/oxygenase (Rubisco) activity **(a)** and CO_2_ fixation rate **(b)** in *Anabaena variabilis* and *Chlorella pyrenoidosa* after 4 days of exposure to 0, 300, and 500 μg L^-1^ Pb (Values represent the mean ± SD of three independent replicates). Asterisks indicate statistically significant differences among treatments, with **** denoting *p* < 0.0001.

In *A. variabilis*, Rubisco activity declined from 93.57 nmol mg^-1^ protein min^-1^ in the control to 41.53 nmol mg^-1^ protein min^-1^ at 300 μg L^-1^ Pb and to 31.58 nmol mg^-1^ protein min^-1^ at 500 μg L^-1^ Pb, corresponding to reductions of 55.6% and 66.3%, respectively, relative to the control. In *C. pyrenoidosa*, Rubisco activity decreased from 94.20 nmol mg^-1^ protein min^-1^ in the control to 27.66 nmol mg^-1^ protein min^-1^ at 300 μg L^-1^ Pb and to 16.15 nmol mg^-1^ protein min^-1^ at 500 μg L^-1^ Pb, representing declines of 70.6% and 82.9%, respectively, compared with the control (Figure 3a).

A similar trend was observed for CO_2_ fixation in *A. variabilis*. CO_2_ fixation declined from 375.65 nmol mg^-1^ DW min^-1^ in the control to 208.85 nmol mg^-1^ DW min^-1^ at 300 μg L^-1^ Pb and to 157.80 nmol mg^-1^ DW min^-1^ at 500 μg L^-1^ Pb, corresponding to decreases of 44.4% and 58.0%, respectively. In *C. pyrenoidosa*, CO_2_ fixation decreased from 340.16 nmol mg^-1^ DW min^-1^ in the control to 167.06 nmol mg^-1^ DW min^-1^ at 300 μg L^-1^ Pb and to 108.10 nmol mg^-1^ DW min^-1^ at 500 μg L^-1^ Pb, equivalent to reductions of 50.9% and 68.2%, respectively. Across all Pb treatments, *A. variabilis* consistently maintained higher Rubisco activity and CO_2_ fixation rates than *C. pyrenoidosa*, although control Rubisco activities were comparable between the two strains (Figure 3b).

### 3.3 Lead stress on ROS generation

**FIGURE 4:**
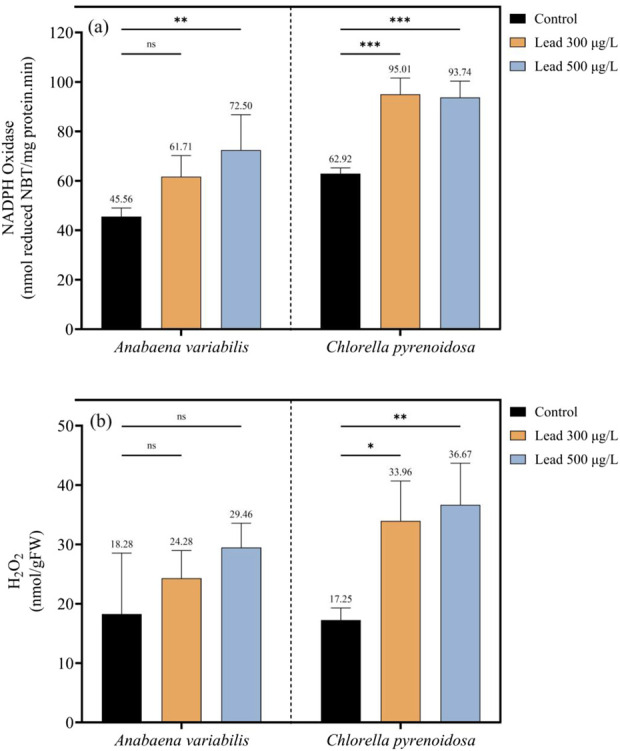
NADPH oxidase activity **(a)** and hydrogen peroxide (H_2_O_2_) content **(b)** in *Anabaena variabilis* and *Chlorella pyrenoidosa* following 4 days of exposure to 0, 300, and 500 μg L^-1^ Pb (Values represent the mean ± SD of three independent replicates). Statistical significance levels are indicated in the figure: “ns” denotes non-significant differences, * indicates p < 0.05, ** indicates p < 0.01, and *** indicates p < 0.001.

In *A. variabilis*, NADPH oxidase activity increased from 45.56 nmol reduced NBT mg^-1^ protein min^-1^ in the control to 61.71 nmol reduced NBT mg^-1^ protein min^-1^ at 300 μg L^-1^ Pb and to 72.50 nmol reduced NBT mg^-1^ protein min^-1^ at 500 μg L^-1^ Pb, corresponding to increases of 35.4% and 59.1%, respectively, relative to the control. In *C. pyrenoidosa*, NADPH oxidase activity increased from 62.92 nmol reduced NBT mg^-1^ protein min^-1^ in the control to 95.01 nmol reduced NBT mg^-1^ protein min^-1^ at 300 μg L^-1^ Pb and to 93.74 nmol reduced NBT mg^-1^ protein min^-1^ at 500 μg L^-1^ Pb, representing increases of 51.0% and 49.0%, respectively, compared with the control (Figure 4a). Consistent with these patterns, H_2_O_2_ content also increased in both strains with increasing Pb concentration. In *A. variabilis*, H_2_O_2_ levels rose from 18.28 nmol g^-1^ FW in the control to 24.28 nmol g^-1^ FW at 300 μg L^-1^ Pb and to 29.46 nmol g^-1^ FW at 500 μg L^-1^ Pb, corresponding to increases of 32.8% and 61.2%, respectively. In *C. pyrenoidosa*, H_2_O_2_ content increased from 17.25 nmol g^-1^ FW in the control to 33.96 nmol g^-1^ FW at 300 μg L^-1^ Pb and to 36.67 nmol g^-1^ FW at 500 μg L^-1^ Pb, representing increases of 96.9% and 112.6%, respectively (Figure 4b). Comparing the two species reveals that *C. pyrenoidosa* consistently exhibited higher NADPH oxidase activity than *A. variabilis* under all Pb treatments. Although H_2_O_2_ concentrations were comparable between species under control conditions, Pb exposure resulted in substantially greater H_2_O_2_ accumulation in *C. pyrenoidosa* than in *A. variabilis*.

### 3.4 Antioxidant defense and oxidative damage

**FIGURE 5:**
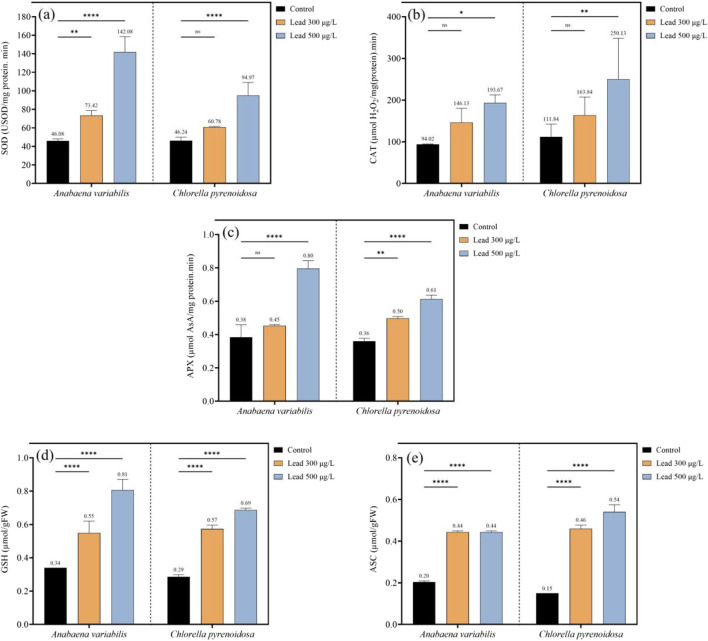
Antioxidant defense responses in *A variabilis* and *Chlorella pyrenoidosa* following 4 days of exposure to 0, 300, and 500 μg L^-1^ Pb, depicting superoxide dismutase (SOD) activity **(a)** catalase (CAT) activity **(b)** ascorbate peroxidase (APX) activity **(c)** reduced glutathione (GSH) content **(d)** and ascorbate (ASC) content **(e)** (Values represent the mean ± SD of three independent replicates). Statistical significance levels are indicated in the figure: “ns” denotes non-significant differences, * indicates p < 0.05, ** indicates p < 0.01, and **** indicates p < 0.0001.

To evaluate the antioxidant defense response, Pb exposure was found to induce significant increases in the activities of SOD, CAT, and APX, as well as in the cellular contents of GSH and ASC, in both algal species examined. In *A. variabilis*, SOD activity increased from 46.08 USOD mg^-1^ protein min^-1^ in the control to 73.42 USOD mg^-1^ protein min^-1^ at 300 μg L^-1^ Pb and to 142.08 USOD mg^-1^ protein min^-1^ at 500 μg L^-1^ Pb, corresponding to relative increases of 59.3% and 208.3%, respectively. In *C. pyrenoidosa*, SOD activity rose from 46.24 USOD mg^-1^ protein min^-1^ in the control to 60.78 USOD mg^-1^ protein min^-1^ at 300 μg L^-1^ Pb and to 94.97 USOD mg^-1^ protein min^-1^ at 500 μg L^-1^ Pb, representing increases of 31.5% and 105.4%, respectively (Figure 5a).

In *A. variabilis*, catalase (CAT) activity increased from 94.02 µmol H_2_O_2_ mg^-1^ protein min^-1^ in the control to 146.13 µmol H_2_O_2_ mg^-1^ protein min^-1^ at 300 μg L^-1^ Pb and to 193.67 µmol H_2_O_2_ mg^-1^ protein min^-1^ at 500 μg L^-1^ Pb, representing increases of 55.4% and 106.0%, respectively. In *C. pyrenoidosa*, CAT activity increased from 111.84 µmol H_2_O_2_ mg^-1^ protein min^-1^ in the control to 163.84 µmol H_2_O_2_ mg^-1^ protein min^-1^ at 300 μg L^-1^ Pb and to 250.13 µmol H_2_O_2_ mg^-1^ protein min^-1^ at 500 μg L^-1^ Pb, corresponding to increases of 46.5% and 123.7%, respectively (Figure 5b).

In *A. variabilis*, ascorbate peroxidase (APX) activity increased from 0.38 µmol AsA mg^-1^ protein min^-1^ in the control to 0.45 µmol AsA mg^-1^ protein min^-1^ at 300 μg L^-1^ Pb and to 0.80 µmol AsA mg^-1^ protein min^-1^ at 500 μg L^-1^ Pb, corresponding to increases of 18.4% and 110.5%, respectively. In *C. pyrenoidosa*, APX activity increased from 0.36 µmol AsA mg^-1^ protein min^-1^ in the control to 0.50 µmol AsA mg^-1^ protein min^-1^ at 300 μg L^-1^ Pb and to 0.61 µmol AsA mg^-1^ protein min^-1^ at 500 μg L^-1^ Pb, representing increases of 38.9% and 69.4%, respectively (Figure 5c).

In *A. variabilis*, the glutathione (GSH) content increased from 0.34 μmol g^-1^ FW in the control to 0.55 μmol g^-1^ FW at 300 μg L^-1^ Pb and to 0.81 μmol g^-1^ FW at 500 μg L^-1^ Pb, corresponding to increments of approximately 61.8% and 138.2%, respectively. In *C. pyrenoidosa*, GSH content rose from 0.29 μmol g^-1^ FW in the control to 0.57 μmol g^-1^ FW at 300 μg L^-1^ Pb and to 0.69 μmol g^-1^ FW at 500 μg L^-1^ Pb, representing increases of 96.6% and 137.9%, respectively (Figure 5d).

In *A. variabilis*, the ASC content increased from 0.20 μmol g^-1^ FW in the control to 0.44 μmol g^-1^ FW at both 300 and 500 μg L^-1^ Pb, representing an increase of 120% at each treatment concentration. In *C. pyrenoidosa*, ASC content increased from 0.15 μmol g^-1^ FW in the control to 0.46 μmol g^-1^ FW at 300 μg L^-1^ Pb and to 0.54 μmol g^-1^ FW at 500 μg L^-1^ Pb, corresponding to increases of 206.7% and 260%, respectively (Figure 5e).

**FIGURE 6:**
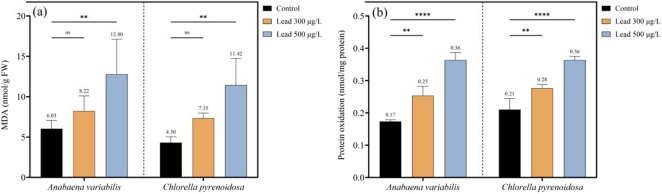
Oxidative damage biomarkers in *Anabaena variabilis* and *Chlorella pyrenoidosa* following 4 days of exposure to 0, 300, and 500 μg L^-1^ Pb: **(a)** malondialdehyde (MDA) content, used as an index of lipid peroxidation, and **(b)** protein oxidation, quantified as protein carbonyl content (Values represent the mean ± SD of three independent replicates). Statistical annotations denote differences among treatments, where “ns” indicates non-significant differences and asterisks denote statistically significant differences (**p < 0.01, ****p < 0.0001).

As a consequence, malondialdehyde (MDA) accumulation and protein oxidation increased in a concentration-dependent manner in both species under Pb exposure. For MDA content, in *A*. *variabilis*, MDA levels increased from 6.03 nmol g^-1^ FW in the control to 8.22 nmol g^-1^ FW at 300 μg L^-1^ Pb and to 12.80 nmol g^-1^ FW at 500 μg L^-1^ Pb, corresponding to increases of 36.3% and 112.3%, respectively, relative to the control. In *C*. *pyrenoidosa*, MDA content rose from 4.30 nmol g^-1^ FW in the control to 7.35 nmol g^-1^ FW at 300 μg L^-1^ Pb and to 11.42 nmol g^-1^ FW at 500 μg L^-1^ Pb, representing increases of 70.9% and 165.6%, respectively (Figure 6a). For protein oxidation, in *A*. *variabilis*, protein carbonyl content increased from 0.17 nmol mg^-1^ protein in the control to 0.25 nmol mg^-1^ protein at 300 μg L^-1^ Pb and to 0.36 nmol mg^-1^ protein at 500 μg L^-1^ Pb, equivalent to increases of 47.1% and 111.8%, respectively. In *C*. *pyrenoidosa*, protein oxidation increased from 0.21 nmol mg^-1^ protein in the control to 0.28 nmol mg^-1^ protein at 300 μg L^-1^ Pb and to 0.36 nmol mg^-1^ protein at 500 μg L^-1^ Pb, corresponding to increases of 33.3% and 71.4%, respectively. Across both oxidative stress markers, *A. variabilis* exhibited higher basal MDA levels than *C. pyrenoidosa*, whereas protein oxidation values at 500 μg L^-1^ Pb were identical between the two species (0.36 nmol mg^-1^ protein) (Figure 6b).

### 3.5 Metabolic adjustment and detoxification mechanisms

#### 3.5.1 Soluble carbohydrates

**FIGURE 7:**
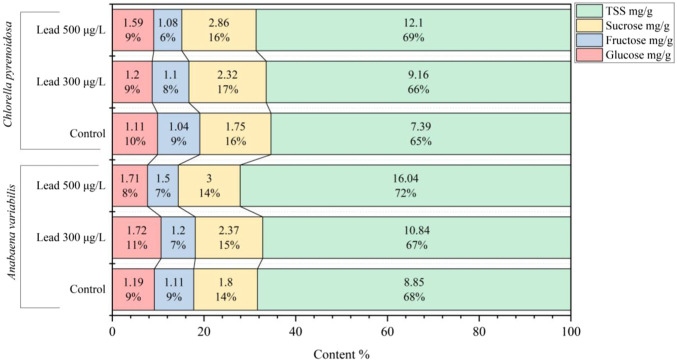
Soluble carbohydrate profiles in *Anabaena variabilis* and *Chlorella pyrenoidosa* following 4 days of exposure to 0, 300, and 500 μg L^-1^ Pb. Each bar represents the absolute content (mg g^-1^) and relative contribution (%) (Values represent the mean of three independent replicates).

The metabolic response to Pb exposure was evaluated by quantifying total soluble sugar (TSS) content and the individual soluble carbohydrates glucose, fructose, and sucrose. The concentrations of TSS, as well as those of glucose, fructose, and sucrose, increased with rising Pb levels in both species. In *A*. *variabilis*, TSS content increased from 8.85 mg g^-1^ in the control to 10.84 mg g^-1^ at 300 μg L^-1^ Pb and to 16.04 mg g^-1^ at 500 μg L^-1^ Pb, corresponding to increases of 22.5% and 81.2%, respectively. In *C*. *pyrenoidosa*, TSS increased from 7.39 mg g^-1^ in the control to 9.16 mg g^-1^ at 300 μg L^-1^ and to 12.10 mg g^-1^ at 500 μg L^-1^ Pb, corresponding to increases of 23.9% and 63.7%, respectively. Sucrose content exhibited a consistent increase with increasing Pb concentration in both strains. In *A. variabilis*, sucrose increased from 1.80 mg g^-1^ in the control to 2.37 mg g^-1^ at 300 μg L^-1^ and to 3.00 mg g^-1^ at 500 μg L^-1^ Pb, representing increases of 31.7% and 66.7%, respectively. In *C. pyrenoidosa*, sucrose content increased from 1.75 mg g^-1^ in the control to 2.32 mg g^-1^ at 300 μg L^-1^ and to 2.86 mg g^-1^ at 500 μg L^-1^ Pb, corresponding to increases of 32.6% and 63.4%, respectively. In *A. variabilis*, glucose content was 1.19 mg g^-1^ in the control, increased to 1.72 mg g^-1^ at 300 μg L^-1^ Pb, and reached 1.71 mg g^-1^ at 500 μg L^-1^ Pb, whereas fructose increased from 1.11 mg g^-1^ in the control to 1.20 mg g^-1^ at 300 μg L^-1^ and 1.50 mg g^-1^ at 500 μg L^-1^ Pb. In *C. pyrenoidosa*, glucose increased from 1.11 mg g^-1^ in the control to 1.20 mg g^-1^ at 300 μg L^-1^ and 1.59 mg g^-1^ at 500 μg L^-1^ Pb, while fructose levels were 1.04, 1.10, and 1.08 mg g^-1^ in the control, 300 μg L^-1^, and 500 μg L^-1^ treatments, respectively (Figure 7).

#### 3.5.2 Amino and organic acids

**FIGURE 8:**
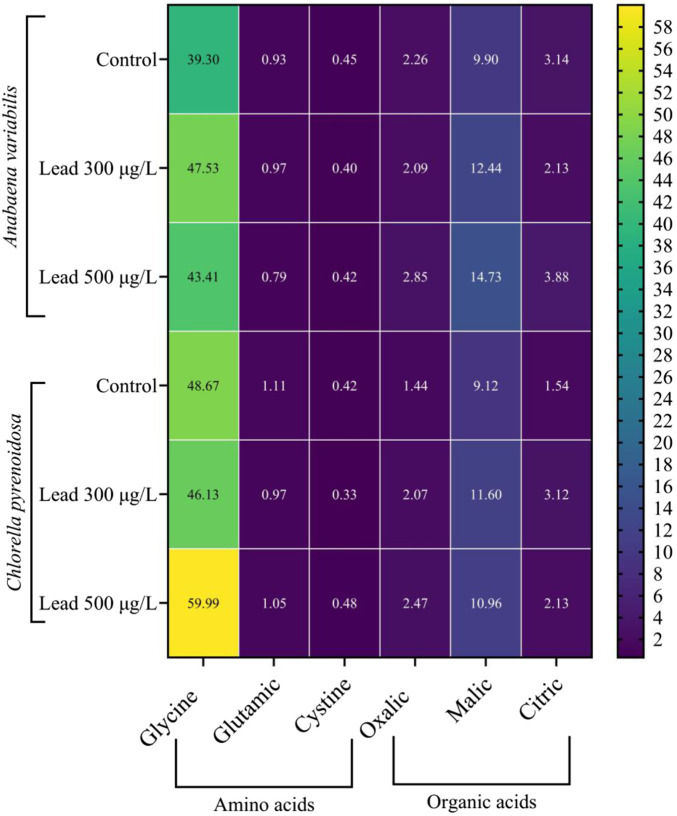
Heatmap illustrating the concentrations of selected amino acids (glycine, glutamic acid, and cystine) and organic acids (oxalic, malic, and citric acids) expressed in mg g^-1^ in *Anabaena variabilis* and *Chlorella pyrenoidosa* following 4 days of exposure to 0, 300, and 500 μg L^-1^ Pb (Values represent the mean of three independent replicates).

In *A. variabilis*, the glycine content increased from 39.30 mg g^-1^ in the control to 47.53 mg g^-1^ at 300 μg L^-1^ Pb, followed by a decline to 43.41 mg g^-1^ at 500 μg L^-1^ Pb, corresponding to a net increase of 10.5% relative to the control at the highest Pb concentration. Glutamic acid exhibited only minor fluctuations, rising from 0.93 mg g^-1^ in the control to 0.97 mg g^-1^ at 300 μg L^-1^ Pb, and subsequently decreasing to 0.79 mg g^-1^ at 500 μg L^-1^ Pb, representing an overall reduction of 15.1% compared with the control. Cystine showed a slight decrease from 0.45 mg g^-1^ in the control to 0.40 mg g^-1^ at 300 μg L^-1^ Pb, followed by a modest increase to 0.42 mg g^-1^ at 500 μg L^-1^ Pb. In *C. pyrenoidosa*, glycine decreased from 48.67 mg g^-1^ in the control to 46.13 mg g^-1^ at 300 μg L^-1^ Pb, then increased markedly to 59.99 mg g^-1^ at 500 μg L^-1^ Pb, corresponding to an increase of 23.3% relative to the control. The glycine concentration at 500 μg L^-1^ Pb in *C. pyrenoidosa* constituted the highest value recorded across all treatments and both species. Glutamic acid decreased from 1.11 mg g^-1^ in the control to 0.97 mg g^-1^ at 300 μg L^-1^ Pb and partially recovered to 1.05 mg g^-1^ at 500 μg L^-1^ Pb. Cystine declined from 0.42 mg g^-1^ in the control to 0.33 mg g^-1^ at 300 μg L^-1^ Pb, followed by an increase to 0.48 mg g^-1^ at 500 μg L^-1^ Pb (Figure 8).

For organic acids in *A. variabilis*, malic acid exhibited the most pronounced Pb-induced increase, rising from 9.90 mg g^-1^ in the control to 12.44 mg g^-1^ at 300 μg L^-1^ Pb and further to 14.73 mg g^-1^ at 500 μg L^-1^ Pb, corresponding to increases of 25.7% and 48.8%, respectively. Citric acid decreased from 3.14 mg g^-1^ in the control to 2.13 mg g^-1^ at 300 μg L^-1^ Pb, then increased to 3.88 mg g^-1^ at 500 μg L^-1^ Pb. Oxalic acid showed a slight decline from 2.26 mg g^-1^ in the control to 2.09 mg g^-1^ at 300 μg L^-1^ Pb, followed by an increase to 2.85 mg g^-1^ at 500 μg L^-1^ Pb. In *C. pyrenoidosa*, oxalic acid increased from 1.44 mg g^-1^ in the control to 2.07 mg g^-1^ at 300 μg L^-1^ Pb and to 2.47 mg g^-1^ at 500 μg L^-1^ Pb, representing increases of 43.8% and 71.5%, respectively. Malic acid increased from 9.12 mg g^-1^ in the control to 11.60 mg g^-1^ at 300 μg L^-1^ Pb and remained elevated at 10.96 mg g^-1^ at 500 μg L^-1^ Pb. Citric acid increased from 1.54 mg g^-1^ in the control to 3.12 mg g^-1^ at 300 μg L^-1^ Pb, an increase of 102.6%, and subsequently declined to 2.13 mg g^-1^ at 500 μg L^-1^ Pb (Figure 8). Collectively, the patterns illustrated in Figure 8 indicate that Pb exposure induced distinct, compound-specific alterations in amino acid and organic acid profiles in both microalgal species, with glycine and malic acid displaying the most consistent and concentration-dependent responses across species.

#### 3.5.3 Fatty acid composition

**FIGURE 9:**
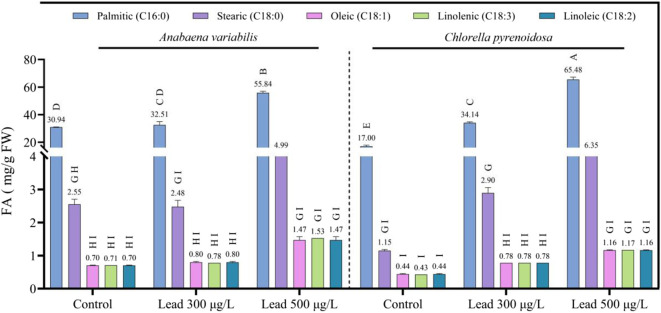
Fatty acid content in *Anabaena variabilis* and *Chlorella pyrenoidosa* after 4 days of exposure to 0, 300, and 500 μg L^-1^ Pb (Values represent the mean ± SD of three independent replicates). Different uppercase letters above the bars denote statistically significant differences among treatment–species combinations according to Tukey’s multiple comparison test (p < 0.05).

In *A. variabilis*, the saturated fatty acid palmitic acid (C16:0) increased from 30.94 mg g^-1^ in the control to 32.51 mg g^-1^ at 300 μg L^-1^ Pb and to 55.84 mg g^-1^ at 500 μg L^-1^ Pb, corresponding to relative increases of 5.1% and 80.5%, respectively. Stearic acid (C18:0) decreased slightly from 2.55 mg g^-1^ in the control to 2.48 mg g^-1^ at 300 μg L^-1^ Pb, but then increased markedly to 4.99 mg g^-1^ at 500 μg L^-1^ Pb, representing a 95.7% increase relative to the control. In *C. pyrenoidosa*, Pb-induced increases in saturated fatty acids were more pronounced. Palmitic acid increased from 17.00 mg g^-1^ in the control to 34.14 mg g^-1^ at 300 μg L^-1^ Pb and to 65.48 mg g^-1^ at 500 μg L^-1^ Pb, reflecting increases of 100.8% and 285.2%, respectively. Stearic acid rose from 1.15 mg g^-1^ in the control to 2.90 mg g^-1^ at 300 μg L^-1^ Pb and to 6.35 mg g^-1^ at 500 μg L^-1^ Pb, corresponding to increases of 152.2% and 452.2%, respectively. Regarding unsaturated fatty acids in *A. variabilis*, oleic acid (C18:1) increased from 0.70 mg g^-1^ in the control to 0.80 mg g^-1^ at 300 μg L^-1^ Pb and to 1.47 mg g^-1^ at 500 μg L^-1^ Pb, corresponding to a 110.0% increase at the highest Pb concentration. Linolenic acid (C18:3) increased from 0.71 mg g^-1^ in the control to 0.78 mg g^-1^ at 300 μg L^-1^ Pb and to 1.53 mg g^-1^ at 500 μg L^-1^ Pb, representing a 115.5% increase relative to the control. Linoleic acid (C18:2) exhibited a trend identical to that of oleic acid, increasing from 0.70 mg g^-1^ in the control to 0.80 mg g^-1^ at 300 μg L^-1^ Pb and to 1.47 mg g^-1^ at 500 μg L^-1^ Pb. In *C. pyrenoidosa*, all three unsaturated fatty acids showed Pb-induced increases in a clear dose-dependent manner. Oleic acid increased from 0.44 mg g^-1^ in the control to 0.78 mg g^-1^ at 300 μg L^-1^ Pb and to 1.16 mg g^-1^ at 500 μg L^-1^ Pb, corresponding to increases of 77.3% and 163.6%, respectively. Linolenic acid increased from 0.43 mg g^-1^ in the control to 0.78 mg g^-1^ at 300 μg L^-1^ Pb and to 1.17 mg g^-1^ at 500 μg L^-1^ Pb, representing an increase of 172.1% relative to the control (Figure 9).

## 4 Discussion

### 4.1 Mechanisms of Pb uptake and bioavailability

Lead accumulation in phototrophic microorganisms, specifically *A. variabilis* and *C. pyrenoidosa*, follows a concentration-dependent biphasic kinetic pattern. This pattern is characterized by an initial rapid surface biosorption phase, succeeded by a slower intracellular sequestration phase. During the current study at a moderate Pb concentration of 300 μg L^-1^, *A. variabilis* demonstrated significantly higher intracellular Pb levels (135.57 μg g^-1^) compared to *C. pyrenoidosa* (52.89 μg g^-1^). This disparity in accumulation can be attributed to the distinct cell-envelope architecture of cyanobacteria, such as *A. variabilis*, which possess extracellular polymeric substance (EPS)-rich surfaces. These surfaces provide extensive, high-capacity metal-binding interfaces, a characteristic consistent with previous findings on cyanobacterial metal interactions (Yadav et al., 2020). Such observations underscore the potential of cyanobacteria to mediate high-affinity surface capture of divalent metal ions, a property highly relevant for the development of surface-engineered bioremediation platforms. However, at an elevated Pb concentration of 500 μg L^-1^, the Pb contents in both species converged, exceeding 200 μg g^-1^. This convergence suggests a saturation of cell wall-associated binding sites, leading to a concomitant shift towards intracellular detoxification mechanisms, particularly evident in the microalga. This transition point is crucial for the rational design of modular sequestration systems in engineered strains, allowing for the partitioning of metal capture between cell wall-associated and intracellular compartments. Furthermore, it provides a conceptual basis for tiered remediation strategies in mixed-species consortia or sequential treatment configurations (Kaur, 2025; Manikandan et al., 2021). The observed metal uptake profiles support the deployment of both strains in Pb-contaminated effluents, with *A. variabilis* exhibiting high bioaccumulation capacity at moderate contamination levels and *C. pyrenoidosa* demonstrating pronounced tolerance and sustained productivity under elevated Pb loads. These findings align with current trends focusing on integrating wastewater valorization with the generation of high-value bioproducts via engineered microbial consortia and phototrophic biorefineries, including process configurations that combine surface adsorption with intracellular sequestration and tailored downstream product streams (Usai et al., 2024). Whereas Pb uptake by microalgae involves both adsorption to the cell surface and active internalization, often via transport routes resembling those used by other divalent cations (e.g., Ca^2+^, Mg^2+^) (Danouche et al., 2021).

### 4.2 Photosynthetic impairment and metabolic reprogramming

Lead exposure exerts a pronounced effect on the photosynthetic apparatus and elicits extensive metabolic reprogramming in phototrophic organisms. Chlorophyll *a* depletion was substantially more severe in *C. pyrenoidosa*, showing a 65.8% reduction at 500 μg L^-1^ Pb, in contrast to *A. variabilis*, which exhibited a 39.1% reduction under the same conditions. This species-specific difference in sensitivity indicates differential susceptibility of the chlorophyll biosynthetic pathways and possibly distinct vulnerabilities of magnesium coordination sites within the pigment-protein complexes of the two taxa. In contrast, carotenoid pools were comparatively stable, resulting in an elevated carotenoid/chlorophyll a ratio (reaching 1.39 at 500 μg L^-1^ in *C. pyrenoidosa*). This response is consistent with a carotenoid-mediated photoprotective strategy, including enhancement of non-photochemical quenching processes, activated in response to impaired electron transport (Fathy et al., 2025; Nowicka, 2022). This mechanistic understanding delineates a concrete target for the rational engineering of photoprotective traits in Pb-stressed phototrophic organisms, intending to sustain continuous bioproduction in real wastewater effluents. In parallel, a concomitant decline in Rubisco activity and CO_2_ fixation rates was detected, with *C. pyrenoidosa* exhibiting a more pronounced reduction (82.9%) relative to *A. variabilis* (66.3%). These observations reveal a primary enzymatic bottleneck in carbon assimilation under Pb stress. The observed pattern highlights key engineering targets, including enhancing Rubisco structural stability or regulatory activation, and/or integrating alternative carbon-concentrating mechanisms to preserve metabolic flux under metal-induced stress conditions.

Pb stress also triggers significant shifts in central metabolism, including carbon, nitrogen, and energy production pathways. The energetic demands of detoxification and reactive oxygen species scavenging necessitate a rerouting of metabolic fluxes towards antioxidant production, metal chelators, and stress-response pathways. This reprogramming often involves a trade-off between growth and survival, particularly under sustained Pb exposure. For instance, a dose-dependent accumulation of soluble sugars was observed in both species, with total soluble sugar content generally higher in *A. variabilis*. The consistent relative proportion of sucrose across treatments supports its primary function as a compatible solute rather than a major carbon storage pool. This osmoprotective strategy aligns with the broader concept of stress-induced carbon reallocation towards protective metabolites, providing a valuable entry point for flux-redistribution approaches in engineered phototrophic systems subjected to metal stress (Miazek and Brożek-Płuska, 2019). Amino acid and organic acid profiles also underwent significant remodeling. A marked increase in glycine was observed in *C. pyrenoidosa* at the highest Pb exposure, consistent with its role as a precursor for glutathione and phytochelatin biosynthesis (Yuksel, 2024). Patterns of glutamic acid accumulation in *A. variabilis* suggest Pb-dependent modulation of the GS/GOGAT pathway activity. Concurrently, concentrations of organic acids such as malic and citric acids increased under Pb treatment, indicating their involvement in metal chelation and vacuolar sequestration. Collectively, these metabolic shifts delineate an integrated reallocation of carbon, nitrogen, and sulfur resources in response to Pb stress.

### 4.3 Redox homeostasis and antioxidant defense networks

Pb exposure elicits a rapid and pronounced surge in reactive oxygen species, characterized by enhanced NADPH oxidase activity and elevated H_2_O_2_ accumulation in both *A. variabilis* and *C. pyrenoidosa*. Notably, *C. pyrenoidosa* exhibited a higher H_2_O_2_ burden at elevated Pb concentrations despite a comparatively lower total Pb uptake. This suggests either an increased peroxide load per unit metal or a reduced capacity for H_2_O_2_ detoxification in *C. pyrenoidosa*. In contrast, the cyanobacterial lineage *A. variabilis* likely exploits lineage-specific redox machineries, such as flavodiiron proteins and peroxiredoxins, to attenuate oxidative bursts, consistent with previously described cyanobacterial redox repertoires (Cavalletti et al., 2022; Parmar et al., 2023). This functional divergence provides a conceptual framework for engineering redox homeostasis in phototrophic systems, either by augmenting peroxide-scavenging capacity or by constructing redox-buffering microdomains that preserve cellular productivity under metal-enriched feedstocks. Enzymatic antioxidant defenses were induced in a concentration-dependent manner in both taxa, with SOD, CAT, and APX all upregulated under Pb stress. CAT induction was particularly pronounced in *C. pyrenoidosa* (123.7% increase at 500 μg L^-1^), aligning with its higher intracellular H_2_O_2_ levels. However, despite robust activation of these antioxidant cascades, markers of oxidative damage to macromolecules remained elevated, suggesting that the detoxification capacity of these systems is exceeded at Pb concentrations ranging from 300 to 500 μg L^-1^. Non-enzymatic antioxidant systems and metal-chelating metabolites were also modulated under Pb stress. Both glutathione and ascorbate pools increased in response to Pb, while glycine accumulation in *C. pyrenoidosa* is consistent with upregulated synthesis of GSH and phytochelatins. Furthermore, *C. pyrenoidosa* displayed elevated cystine levels at the highest Pb dose, indicative of intensified sulfur allocation toward phytochelatin-mediated sequestration of Pb. Collectively, these metabolic shifts delineate actionable targets for metabolic engineering to reinforce intracellular metal chelation and vacuolar sequestration, which represent central mechanisms in Pb tolerance and are highly relevant for designing microalgal platforms for Pb bioremediation and metal-resilient bioprocesses.

### 4.4 Membrane remodeling and subcellular sequestration

Lead stress induces significant membrane remodeling and subcellular adaptations in phototrophic microorganisms. A paradoxical enrichment of both saturated (e.g., C16:0, C18:0) and polyunsaturated fatty acids (e.g., C18:1, C18:2, C18:3) was observed. This concurrent upregulation is consistent with a compartmentalized membrane remodeling strategy. Increased saturation in the plasma membrane likely promotes rigidification, thereby restricting Pb^2+^ influx, while the enrichment of polyunsaturated species in the thylakoid membranes preserves sufficient fluidity to sustain photosynthetic electron transport. This proposed spatial partitioning preferential accumulation of saturated fatty acids in the plasma membrane and polyunsaturated fatty acids in thylakoid membranes constitutes a testable framework for lipid engineering in phototrophic organisms exposed to heavy metal stress (Zhukov and Volkov, 2025). It also provides a rational basis for developing culture conditions or targeted genetic modifications aimed at enhancing membrane robustness under metal exposure. The magnitude of this remodeling has significant bioprocess implications. The more pronounced lipid remodeling observed in *C. pyrenoidosa* suggests a higher energetic investment in membrane adaptation. However, this investment also confers greater robustness of photosynthetic metabolism under Pb^2+^ stress, an advantageous trait for maintaining biomass productivity in integrated remediation-bioproduct processes. These results are directly relevant to strain selection and process optimization in photobioreactor systems treating Pb-contaminated effluents, where membrane lipid composition can modulate membrane integrity, stress signaling pathways, and ultimately product yields. The microalgal cell wall and plasma membrane serve as the initial contact points for Pb. Adsorption to negatively charged cell surfaces and interactions with wall polysaccharides and lipids determine the initial uptake and intracellular distribution.

### 4.5 Engineering implications for phycoremediation and biomonitoring

Microalgae present a dual capacity for mitigating lead contamination: (i) the efficient removal of Pb from polluted aquatic systems via cellular uptake, intracellular sequestration, and surface adsorption, and (ii) their deployment as sensitive biosensors, based on quantifiable perturbations in photosynthetic efficiency, cellular redox homeostasis, and metabolomic signatures. Targeted bioengineering interventions that enhance the synthesis and affinity of metal-binding ligands, optimize Pb-detoxification and compartmentalization pathways, and maintain or improve photosynthetic performance under Pb-induced stress can synergistically increase both metal accumulation efficiency and biomass productivity. Collectively, these advances underpin the development of scalable and economically viable phycoremediation approaches for Pb-contaminated environments (Ankit and Korstad, 2022). Species selection is a critical consideration; a judicious triage among green algae, diatoms, and cyanobacteria should account for factors such as cell wall composition, uptake kinetics, baseline antioxidant capacity, and the capacity for vacuolar sequestration. Eco-physiological studies coupled with targeted genetic engineering can optimize strains for specific Pb-contaminated habitats (Ankit and Korstad, 2022; Das et al., 2022). The documented accumulation of soluble sugars, amino acids (particularly glycine), and organic acids indicates the activation of a coordinated osmotic-adjustment and detoxification program. Organic acids are likely to participate in extracellular or vacuolar chelation of Pb, while compatible solutes contribute to the stabilization of proteins and lipid bilayers under combined oxidative and osmotic stress (Osmolovskaya et al., 2018). These experimental outcomes can be directly translated into specific engineering targets and design rules. Potential interventions include: (i) increasing cell-surface binding capacity and establishing controlled Pb transport routes; (ii) reinforcing redox-buffering networks and compartmentalized sequestration; (iii) reconfiguring GSH/phytochelatin and organic-acid biosynthetic and transport pathways to enhance intracellular Pb sequestration and efflux control; and (iv) deploying lipid-remodeling programs to maintain photosynthetic performance under metal-induced stress. Collectively, these strategies are congruent with the agenda on mechanism-guided engineering of phototrophic chassis for environmental remediation and sustainable bioproduction. The complementary physiological and metabolic responses observed in *A. variabilis* and *C. pyrenoidosa* provide a strong rationale for designing engineered consortia. Such consortia could integrate efficient cell-surface biosorption with adaptable metabolic fluxes, thereby optimizing Pb uptake, intracellular detoxification, and downstream product recovery. This concept aligns with the growing interest in architecting synthetic phototrophic–heterotrophic communities for wastewater treatment and the generation of value-added bioproducts under stress conditions. The complementary behavior of the two phototrophs suggests a practical route for combined Pb remediation. *Anabaena variabilis* appears better suited for the initial treatment stage, where rapid Pb capture at moderate contamination levels is required, whereas *C*. *pyrenoidosa* appears more suitable for downstream polishing under higher residual stress because of its stronger metabolic plasticity and acclimatory capacity. Together, these traits support the feasibility of either sequential treatment configurations or mixed-species consortia for Pb-contaminated effluents, although pilot-scale validation under continuous-flow conditions is still needed.

## 5 Conclusion

This study provides an assessment of the responses of the phototrophic microorganisms *A. variabilis* and *C. pyrenoidosa* to Pb-induced stress. The results demonstrate that Pb exposure elicits a concentration-dependent reprogramming of cellular physiology, encompassing metal uptake, carbon assimilation, redox homeostasis, osmotic regulation, and membrane lipid architecture. Collectively, these observations support the view that Pb toxicity constitutes a systems-level phenomenon driven by tightly interconnected physiological and metabolic adjustments. A major contribution of this work is the delineation of distinct, species-specific Pb management strategies. *Anabaena variabilis* exhibited greater Pb accumulation at moderate concentrations (300 μg L^-1^), indicative of efficient extracellular biosorption, whereas *C. pyrenoidosa* showed enhanced accumulation at higher concentrations (500 μg L^-1^), consistent with a greater reliance on intracellular sequestration and associated tolerance mechanisms. This functional divergence underscores their complementary applicability across a gradient of contamination levels and provides a rationale for the deployment of mixed-species assemblages in bioremediation. Pb exposure substantially impaired the photosynthetic apparatus in both phototrophs, as reflected by decreases in chlorophyll a content, Rubisco activity, and CO_2_ fixation rates. The more pronounced inhibition in *C. pyrenoidosa* indicates heightened sensitivity of its photosynthetic machinery. Nonetheless, the relative stability of carotenoids and the increased carotenoid-to-chlorophyll ratio point to the activation of photoprotective mechanisms that partially attenuate photodamage, emphasizing the critical role of pigment stoichiometry and sustained carbon assimilation under metal stress. Oxidative stress emerged as a central component of Pb toxicity, evidenced by elevated reactive oxygen species production and concomitant lipid peroxidation and protein oxidation. Although both species upregulated their antioxidant defense systems, these responses were inadequate to fully alleviate oxidative injury at higher Pb concentrations. This finding indicates that tolerance is governed by the dynamic balance among ROS generation, detoxification capacity, and cellular repair or sequestration processes. Metabolic profiling further revealed a coordinated reconfiguration of primary and secondary metabolism under Pb stress, including: (i) accumulation of soluble sugars contributing to osmoprotection; (ii) alterations in amino acid and organic acid pools that support metal chelation and the biosynthesis of antioxidant molecules; and (iii) remodeling of fatty acid composition to stabilize membrane structure and modulate metal influx. Together, these adjustments demonstrate that acclimation to Pb stress necessitates integrated regulation of redox balance, osmotic stability, and membrane integrity. Overall, this study advances mechanistic understanding of Pb toxicity in phototrophs by explicitly linking metal accumulation patterns with oxidative stress dynamics and metabolic adaptation in a comparative framework. From an applied perspective, *A. variabilis* appears particularly suitable for Pb sequestration under moderate contamination scenarios, whereas *C. pyrenoidosa* displays greater metabolic plasticity under elevated Pb stress. Their complementary functional traits support the rational design of optimized multispecies consortia for phycoremediation. Furthermore, the identified physiological and biochemical responses constitute robust biomarker candidates for monitoring Pb stress in aquatic ecosystems, thereby providing a foundation for improved environmental assessment and targeted biotechnological applications.

## Data Availability

The original contributions presented in the study are included in the article/supplementary material, further inquiries can be directed to the corresponding author.
